# Muscle Activation Characteristics of the Front Leg During Baseball Swings with Timing Correction for Sudden Velocity Decrease

**DOI:** 10.1371/journal.pone.0124113

**Published:** 2015-04-28

**Authors:** Yoichi Ohta, Hiroki Nakamoto, Yasumitsu Ishii, Sachi Ikudome, Kyohei Takahashi, Norihiro Shima

**Affiliations:** 1 Faculty of Health and Medical Sciences, Department of Sports and Health Sciences, Aichi Shukutoku University, Aichi, Japan; 2 Faculty of Sports Humanities and Applied Social Science, Department of Physical Education, National Institute of Fitness and Sports in Kanoya, Kagoshima, Japan; 3 Department of Sports Science, Japan Institute of Sports Sciences, Tokyo, Japan; 4 Faculty of Liberal Studies, National Institute of Technology, Kumamoto College, Kumamoto, Japan; 5 School of Sport and Health Science, Tokai Gakuen University, Aichi, Japan; University of Manchester, UNITED KINGDOM

## Abstract

This study aimed to clarify the activation characteristics of the vastus lateralis muscle in the front leg during timing correction for a sudden decrease in the velocity of a target during baseball swings. Eleven male collegiate baseball players performed coincident timing tasks that comprised constant velocity of 8 m/s (unchanged) and a sudden decrease in velocity from 8 to 4 m/s (decreased velocity). Electromyography (EMG) revealed that the muscle activation was typically monophasic when responding unchanged conditions. The type of muscle activation during swings in response to decreased velocity condition was both monophasic and biphasic. When biphasic activation appeared in response to decreased velocity, the impact time and the time to peak EMG amplitude were significantly prolonged and the timing error was significantly smaller than that of monophasic activation. However, the EMG onset from the target start was consistent both monophasic and biphasic activation in response to conditions of decreased velocity. In addition, batters with small timing errors in response to decreased velocity were more likely to generate biphasic EMG activation. These findings indicated that timing correction for a sudden decrease in the velocity of an oncoming target is achieved by modifying the muscle activation characteristics of the vastus lateralis muscle of front leg from monophasic to biphasic to delay reaching peak muscle activation and thus prolong impact time. Therefore, the present findings suggests that the extent of timing errors in response to decreased velocity is influenced by the ability to correct muscle activation after its initiation rather than by delaying the initiation timing of muscle activation during baseball swings.

## Introduction

The timely interception of a high-velocity object such as a ball using batting tools is one of the most difficult perceptual motor acts that a human can perform [1]. A bat must be swung to a precise spatial position and the timing must be simultaneously adjusted based on a temporal prediction at least within 400–800 ms in baseball batting [2–4]. When a batter can precisely predict the moment at which a thrown ball arrives, it can be hit by swinging the bat to the impact position at the appropriate time. Furthermore, skilled cricket players can alter the swing of their bats within ~ 190 ms of the moment when a ball bounces oddly [5]. This finding indicates that batters can rapidly adjust and correct the swing and its timing to unexpected perturbations of ball trajectories.

Correcting swing timing due to unexpected changes in the status of a target such as in velocity and/or impact point is associated with adjusting the activities of upper limb muscles [6]. However, whether the control of lower limb muscle activation is related to timing correction due to unexpected changes in velocity remains unclear. Front leg movement (time of landing and time from landing to weighting) is closely associated with controlling the timing required to hit a ball [7]. The landing of the front leg on the ground and the start of the front leg knee extension occur at about the same time [8]. In the phase of landing to weighting, the knee extension velocity increases with the increases in ground reaction force of the front leg [8]. Moreover, the knee extensor muscle of the front leg is greatly activated during the phase from landing to shifting the body weight onto the front leg [9]. Thus, the controlling knee extensor muscle activities (e.g. onset timing, time to peak, and peak amplitude) in the front leg may be important for the timing control of hitting an oncoming ball. However, no systematic studies have shown how this occurs due to unexpected changes in the target during the baseball swinging motion. Therefore, we hypothesized that due to unexpected changes in target status, swing timing is corrected by modifying knee extensor muscle activation strategy in the front leg.

The muscle activation strategy is clarified based on analysis of the shape of the electromyographic (EMG) patterns [10–12]. The EMG pattern was characterized by monophasic or multi-phasic (included in biphasic) activation patterns [11, 12]. Thus, classifying mono- or biphasic activation is a useful analysis method for examining the muscle activation strategy in the baseball swinging movement. Moreover, understanding the control and coordination required to hit a baseball can help to advance the theoretical understanding of coordinative structures in sport performance [7]. Thus, the goal of the present study is to deepen the theoretical understanding of coordinative structures in sports performance through the clarification of the characteristics of a muscle activation strategy associated with timing correction due to unexpected velocity changed condition.

The present study aimed to clarify the muscle activation characteristics of the front leg associated with timing correction due to unexpected changes in the target during baseball swings using a batting simulator. Moreover, we also investigated the muscle activation characteristics of the front leg of batters with small timing errors since timing correction ability differs among expert baseball batters [6, 13].

## Methods

### Participants and ethics statement

Eleven male collegiate baseball players (21.1 ± 0.8 years; 172.5 ± 7.3 cm; 65.7 ± 5.7 kg) with 8 to 12 years of experience participated in this study. They generally spent 15 h/week in baseball training and regularly participated in competitions. The present study proceeded in accordance with the Declaration of Helsinki and was approved by the Ethics Committee of the National Institute of Fitness and Sports in Kanoya, Japan. All participants received a full explanation of the objectives of the investigation and were informed of the experimental procedures in advance of the study. All of the players provided written informed consent to participate in the study.

### Experimental apparatus and task

The experimental device (batting simulator) consisting of an AO-5N horizontal electronic trackway (length, 4 m; height, 60 cm) with 200 light-emitting diodes (LEDs) that simulated the linear motion of an object (Applied Office Co. Ltd., Tokyo, Japan) has been used in several studies [6, 13]. The LEDs were quickly turned on and off in sequence so that participants could clearly perceive the continuous motion of an approaching target as a flashing light. The participants performed a coincident timing task that comprised swinging a bat (length, 86 cm; weight, 850 g) at the moment of arrival of an apparently moving target that ran on a straight trackway. The moving target was displaced from one end of the trackway at a constant velocity (8 m/s; unchanged condition) after the presentation of a 3-s visual light stimulus from five LEDs. In another trial, the target velocity suddenly decreased by 250 ms after starting to move (decreased velocity condition, DV). These manipulations of target velocity before the target arrived at the impact point (above the edge of the trackway) required the participants to temporally correct the swing of the bat. The velocity change was set at 50% (from 8 to 4 m/s), which is above the threshold value that is generally deemed sufficient to allow the perception of a variation [14, 15]. The total durations of target presentation in response to unchanged and DV were 500 and 750 ms, respectively. The total duration of target presentation in response to the unchanged condition creates situations that a baseball batter would generally face during an actual baseball game [2–4].

### Experimental procedure

The participants first stood beside a standard home plate that was placed on the floor just under the front edge of the trackway. They repeated the experimental procedure in response to unchanged and DV until they became familiarized. The participants were required to swing to meet the arrival of the target at the impact point in response to unchanged and DV. Moreover, we required the participants to minimize timing errors between the target and the bat arriving at the impact point. The participants were given verbal feedback concerning temporal errors. The participants subsequently four sets of six swings at maximal effort for a total of 24 swings for coincident timing tasks. The sets included two equivalent conditions that changed in random order of a 50% chance of a velocity decrease. The set interval was 3–5 min.

### Detection and analysis of experimental signals

Temporal errors for coincident timing tasks were calculated from the difference between the total duration (500 or 750 ms) and the moment that the bat arrived at the impact point (above the edge of the trackway) with respect to the start of the target movement. Impact time was measured using a photoelectric tube system installed at the edge of the center and the outside trackway using an off signal that was sent when a bat crossed this path. Temporal errors were evaluated as the absolute temporal error (ATE), which is an index of the accuracy of the coincident timing performance of a baseball bat swung during a task involving a temporal change [6, 13]. Trials having an ATE above or below the mean ± 2 standard deviations (SD) were excluded from all subsequent analyses. The electrical signals from the photoelectric tube system and the batting simulator (at release of the moving target and at 250 ms thereafter) were recorded using Chart 5.4.2 software (AD Instruments Pty. Ltd., Bella Vista, Australia) via a Power-Lab 16sp analog-to-digital (A/D) converter (AD Instruments) at a sampling rate of 2 kHz. These stored electronic signals served as a trigger to synchronize the electromyographic (EMG) data.

The EMG signal was measured from the vastus lateralis muscle (VL) of the front leg using a WEB-7000 telemeter system with a 15–500 Hz band-pass filter (Nihon Kohden, Tokyo, Japan). The telemeter electrodes were used that is built-in electrode and telemeter (SB-150H, Nihon Kohden, Tokyo, Japan). The electrode was a silver material, and the inter-electrode distance was 1 cm. The skin surface was cleaned with alcohol and rubbed with sandpaper. Electrodes were located on the muscle belly and aligned parallel to muscle fibers. The EMG signals were recorded using a computer via an A/D converter at a sampling rate of 2 kHz. The EMG signals were rectified and integrated (time constant, 50 msec) before the analysis of EMG signals. EMG signals were recorded on-line to a computer (Chart 5.4.2 software, AD Instruments, Australia) via an A/D convertor (Power-Lab 16sp, AD Instruments, Australia) at a sampling rate of 1 kHz.

The onset of EMG activity from the target was taken as the time point at which the EMG amplitude exceeded a threshold, and after EMG amplitude remained above that level for at least 25 ms [12, 16, 17]. The threshold level was set to a mean background activity that calculated 200 ms of muscle activity within 250 ms after the target started plus 4 standard deviations. Complex skilled multiple joint movements were assessed, and since activity in the VL often occurs before a main burst, the onset of EMG activity was more difficult to determine compared with single-joint movements. When a clear error was found to determine EMG onset using the above method, EMG onset was determined by visual inspection of raw and integrated EMG signals [18].

**Fig 1:**
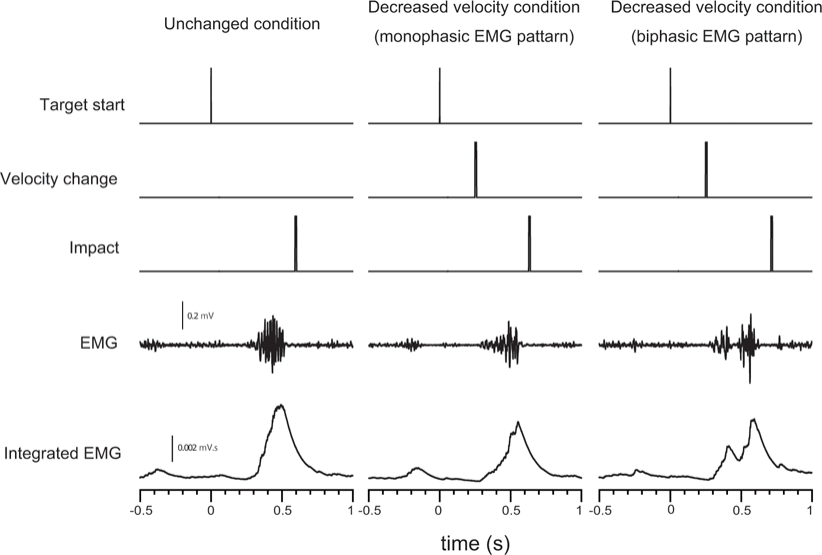
Example of time course of EMG signals in vastus lateralis muscle of front leg. Monophasic and biphasic EMG activation of vastus lateralis muscle of front leg of one participant in response to unchanged and decreased velocity conditions, respectively. Target start time was 0 s.

In response to DV, single (monophasic) and double (biphasic) peak EMG activation was determined by visual inspection (Fig 1). Biphasic EMG activation was defined as a clear reduction in EMG amplitude after the first peak followed by a clear increase. The time to peak EMG amplitude (TP) from the target start and peak EMG amplitude (EMGmax) for each trial were measured. Latency between the onset of EMG activation and the time to peak was measured. During biphasic EMG activation, TP, EMGmax, and latency were measured from the first and second peaks. The EMGmax was normalized to the maximum EMG amplitude determined during isometric maximal voluntary contraction (MVC) and is expressed as a ratio (%) of the MVC (%MVC).

### Statistical analysis

All data are presented as means ± SD. Differences in mean values among the unchanged condition and monophasic and biphasic types of EMG activation were determined using a one-way repeated measures ANOVA for all parameters. The mean values in all trials in response to DV were excluded from statistical analysis. Significant main effect parameters were assessed by multiple comparison tests using Bonferroni correction. Pearson product moment correlation coefficients (r) were calculated to determine correlations between parameters using inter-subject data (n = 11). Data including effect size indexes (*η*_p_^2^) were statistically analyzed using SPSS for Windows, version 22.0 (IBM, New York, NY, USA). The effect size index (*d*) was calculated using Cohen’s *d* index [19, 20].

## Results

### Coincident timing performance and impact time

**Fig 2:**
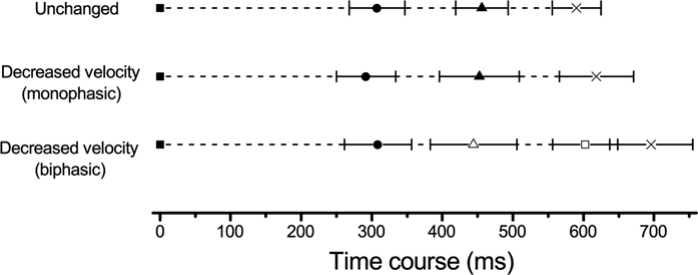
Outline of elapsed time from moment that target started to move until impact in response to unchanged and decreased velocity. Data are shown as means and SD. Target start (■), Onset of EMG activation (●), time to peak EMG amplitude in response to unchanged condition and monophasic EMG pattern in decreased velocity condition (▲), time to peak EMG amplitude of first (△) and second (□) peaks in response to biphasic EMG pattern in decreased velocity condition, impact time (×).

Table 1 shows the means and standard deviations (SD) of the ATE and the impact time for all trials in response to unchanged condition and for swings with monophasic and biphasic EMG activation in response to DV. One-way ANOVA of ATE data indicated a significant main effect (*F*_2, 20_ = 6.01, *p* = 0.009, *η*_p_^2^ = 0.37). The mean ATE was significant lower for biphasic, than for monophasic EMG activation (*p* = 0.002, d = 1.45). A significant main effect was found for impact time (*F*_2, 20_ = 24.17, *p* < 0.001, *η*_p_^2^ = 0.70). The mean impact time was significant longer for biphasic, than for monophasic EMG activation (*p* = 0.005, d = 1.39) and the unchanged condition (*p* < 0.001, d = 2.20). Outline of elapsed time from moment that target started to move until impact in response to unchanged and decreased velocity is shown in Fig 2.

**Table 1:** Absolute temporal error (ATE) and impact time from when a target starts to move (impact time) in response to unchanged and decreased velocity conditions.

	Unchanged	Decreased velocity		
		Monophasic	Biphasic	Both
**ATE (ms)**	92.1 ± 34.6	133.1 ± 48.7*	74.3 ± 30.7	108.5 ± 41.6
**Impact time (ms)**	590.1 ± 34.6*	618.4 ± 52.8*	696.0 ± 58.7	656.6 ± 61.1

*Significantly different from biphasic values (p < 0.01). Both values are shown as means of all trials in response to decreased velocity. Both values were excluded from statistical analysis.

### EMG profiles

#### EMG onset, time to peak, latency between onset of EMG activation and time to peak, and peak EMG amplitude

Table 2 shows the means and SD of the onset of EMG activation from the target starting to move in response to unchanged condition and for swings with monophasic and biphasic EMG activation in response to DV. One-way ANOVA of these data indicated no significant main effect (*F*_2, 20_ = 1.95, *p* = 0.168, *η*_p_^2^ = 0.16).

**Table 2:** Electromyographic onset from target start (EMG onset), time to peak EMG amplitude from target start (TP), latency time between onset of EMG and time to peak (Latency), and peak EMG amplitude (EMGmax) in response to unchanged and decreased velocity conditions.

	Unchanged	Decreased velocity			
		Monophasic	Biphasic (first peak)	Biphasic (second peak)	Both
**EMG onset (ms)**	307.3 ± 39.4	291.8 ± 41.9	307.4 ± 46.3	--	298.8 ± 43.9
**TP (ms)**	456.0 ± 37.0*	452.9 ± 56.5*	446.3 ± 61.8*	599.8 ± 50.5	514.9 ± 60.3
**Latency (ms)**	148.8 ± 22.7*	161.0 ± 46.9*	138.7 ± 49.7*	292.4 ± 61.6	216.1 ± 62.6
**EMGmax (%)**	126.8 ± 55.2^†^	116.0 ± 67.1	77.6 ± 39.6	106.5± 54.5	111.5± 61.0

A significant main effect was found for EMGmax (*F*_3, 30_ = 9.15, *p* < 0.001, *η*_p_^2^ = 0.47), which was significantly lower for the first peak of biphasic EMG activation than unchanged condition (*p* = 0.007, *d* = 1.03).

Significantly different from biphasic second and first peak values:

*p < 0.01

^†^ *p* < 0.01, respectively.

Both values are shown as means of all trials in response to decreased velocity. Both values were excluded from statistical analysis.

Table 2 also shows the means and SD of the time required to time to peak EMG amplitude (TP), latency from the onset of EMG activation until time to peak (Latency), and the peak EMG amplitude (EMGmax) from the moment that the target started to move for all trials in response to unchanged condition and for swings with monophasic and biphasic (first and second peak) EMG activation in response to DV.

One-way ANOVA of the TP data indicated a significant main effect (*F*_3, 30_ = 25.56, *p* < 0.001, *η*_p_^2^ = 0.71). The TP was significantly longer for the second peak during biphasic EMG activation than the unchanged condition (*p* < 0.001, *d* = 3.55), monophasic EMG activation (*p* = 0.001, *d* = 2.74) and the first peak of biphasic EMG activation (*p* < 0.001, *d* = 2.72).

A significant main effect was found for latency (*F*_3, 30_ = 33.80, *p* < 0.001, *η*_p_^2^ = 0.77). Latency was significantly longer for the second peak of biphasic EMG activation than that in response to unchanged condition (*p* < 0.001, *d* = 3.10), monophasic EMG activation (*p* < 0.001, *d* = 2.40), and the first peak of biphasic EMG activation (*p* < 0.001, *d* = 2.75).

### Inter-subject correlations between ATE and EMG profiles

**Fig 3:**
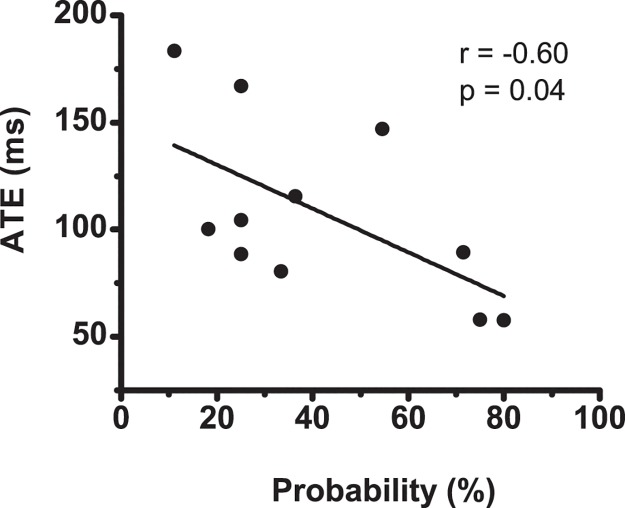
Relationship between ATE and probability. Relationship between absolute temporal error (ATE) in response to decreased velocity condition and probability that biphasic EMG pattern will appear in response to this condition.

**Fig 4:**
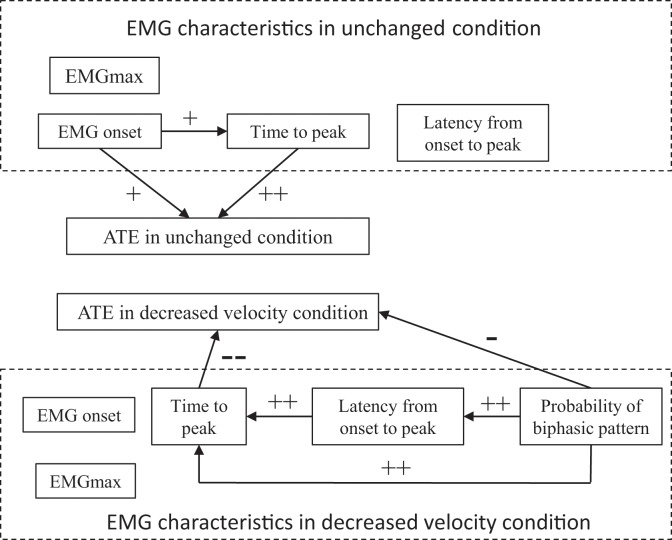
Schematic diagram of relationships between absolute temporal error (ATE) and electromyographic (EMG) characteristics. Relationships: +, positive, *p* < 0.05; ++, positive, *p* < 0.01; --, negative, *p* < 0.01; -, negative, *p* < 0.05.

Table 3 shows inter-subject correlations between the ATE and EMG profiles in the 11 participants. The mean probability of a biphasic EMG pattern appearing in response to DV was 41.4 ± 24.6%. Inter-subject correlation was significant between the probability of a biphasic EMG pattern appearing and the ATE in response to DV (*r* = -0.604, *p* = 0.049; Fig 3). Fig 4 shows a schematic diagram of these relationships.

**Table 3:** Inter-subject correlations (r) between parameters (n = 11).

	ATE (U)	ATE (DV)	ATE (All)	TP (U)	TP (DV)	Onset (U)	Onset (DV)	Latency (U)	Latency (DV)	Max (U)	Max (DV)
**Prob**	-.027	-.604*	-.684*	-.156	.774^†^	-.207	-.110	.104	.823^†^	.010	.074
**ATE (U)**	-	-.513	.348	.914^†^	.386	.653*	.597	.357	-.047	.179	.340
**ATE (DV)**		-	.626*	-.321	-.802^†^	-.138	-.401	-.283	-.491	-.027	-.209
**ATE (All)**			-	.479	-.526	.442	.105	.015	-.580	.132	.080
**TP (U)**				-	.277	.824^†^	.718*	.201	-.236	.171	.251
**TP (DV)**					-	.202	.310	.102	.746^†^	.220	.350
**Onset (U)**						-	.845^†^	-.388	-.398	.192	.254
**Onset (DV)**							-	-.294	-.402	.266	.369
**Latency (U)**								-	.304	-.054	-.031
**Latency (DV)**									-	.026	.078
**Max (U)**										-	.950^†^

ATE, absolute temporal error; U, unchanged condition; DV, decreased velocity; Latency, latency time from onset of EMG activation to time to peak; Max, Peak EMG amplitude; Onset, onset of EMG activation from target start; Prob, probability that biphasic EMG will appear in response to decreased velocity; TP, time to peak from target start.

*p < 0.05

^†^ *p* < 0.01.

## Discussion

Previous analyses of ground reaction force and centers of pressure during baseball batting have revealed that batters step with their front leg in the direction of the pitcher to initiate a batting [21] and then shift their weight to ground the front leg and generate hip and trunk rotation [22–24]. Therefore, considering changes in ground reaction force and centers of pressure during baseball batting [21–24], EMG activation of the VL muscle after the target started to move in the present study reflects muscle activity associated with the force loading to a front leg after landing during the batting.

In response to unchanged target velocity condition, EMG activation was typically monophasic (Fig 1), indicating that the force generated from the front leg becoming grounded is generated by a chain of events. Monophasic and biphasic EMG activation was evident in response to decreased target velocity condition (DV) (Fig 1). The monophasic EMG activation response to DV might reflect that a muscle activation strategy similar to the unchanged condition was used. In contrast, biphasic EMG activation in response to DV might have been caused by delaying the weight shift onto the front leg and/or the generation of force by grounding the front leg because batters must delay the weight shift onto the front leg to hit slow balls [7]. The monophasic and biphasic types of EMG activation during swings might reflect a difference in the muscle activation strategy required to swing effectively between unchanged and DV. Analysis of the effects of monophasic and biphasic EMG activation on coincident timing performance would clarify muscle activation strategy associated with timing correction. Therefore, we discuss the effects of monophasic and biphasic EMG activation of VL muscle in front leg on coincident timing performance during baseball swing in detail.

If information about the velocity (slow or fast) of a pitch is not predicted, the front leg would consistently land on the ground at the same time when hitting both slow and fast pitches; hence batters prepare movements to hit fast pitches [7]. The EMG onset of VL from the target start in the present study was consistent for both unchanged and DV that included monophasic and biphasic EMG activation (Table 2 and Fig 2). These findings suggested that batters prepared the onset timing of muscle activation to match the timing of target arrival by means such as early impact timing in response to unchanged condition rather than DV. Moreover, impact time did not significantly differ between the unchanged and DV with monophasic EMG activation (Table 1 and Fig 2). The similar impact time indicated that when EMG activation was monophasic, the batter did not correct timing through a decrease in target velocity but swung the bat with the same timing as that in response to unchanged condition. In addition, the time to peak EMG amplitude (TP) did not significantly differ between unchanged condition and monophasic EMG activation in response to DV (Table 2 and Fig 2). These findings indicated that when a batter did not correct timing through a decrease target velocity, the muscle activation characteristics from onset to peak were similar between unchanged condition and DV. Namely, the muscle activation strategy in the VL from activation onset to peak in response to unchanged condition also appeared when the batter did not react to a decrease in target velocity. Therefore, batters performed coincident timing tasks based on a muscle activation strategy that matched the timing of the swing in response to unchanged condition.

Impact time was significantly delayed in response to DV during biphasic, compared with monophasic EMG activation (Table 1 and Fig 2), and the timing error was significantly smaller than that of monophasic EMG activation (Table 1). The EMG onset did not significantly differ between the biphasic and monophasic EMG activation. However, the TP was significantly longer for biphasic, than for monophasic EMG activation (Table 2 and Fig 2). The prolonged TP during biphasic EMG activation might have resulted from a delay in the forward weight shift and/or peak force generation due to grounding the front leg. Moreover, the first TP during biphasic EMG activation did not significantly differ from that in response to unchanged condition and monophasic EMG activation. However, the EMG amplitude was lower for the first peak of biphasic EMG activation than for that in response to unchanged condition and monophasic EMG activation (Table 2). These results suggested that batters correct the activation amplitude and latency after initiating activation but not the activation timing (onset) based on unchanged condition to react to a target velocity decrease and then re-activate the muscles; that is, muscle activation becomes biphasic. Therefore, the present findings suggests that batters correct timing using a biphasic muscle activation strategy that is intended to delay peak muscle activation to prolong impact time in adaptation to a decrease in target velocity. This may imply that the form that can coordinate force of front leg is good.

The mean EMG onset of biphasic EMG activation in response to DV was 307.4 ± 46.3 ms (target velocity started to decrease 250 ms after starting), indicating that muscle activation was initiated after the target velocity decreased. However, since batting reaction time is approximately 200 ms [5, 25, 26], the motor plan associated with muscle activation was probably not started after confirming a decrease in velocity. Because the first peak amplitude during biphasic EMG activation was significantly lower than the peak EMG amplitude in response to unchanged condition and monophasic EMG activation, a monophasic muscle activation strategy can be modified to a biphasic type at the latest, by the first peak at ~190 ms after velocity decreases. That is, it is suggested that batters apparently correct timing by reorganizing the initiated motor command using a muscle activation strategy based on unchanged condition after a decrease in target velocity is confirmed.

A study of arm movement dynamics found that when an end-point is changed after arm movement initiated, the aiming movement is reorganized based on a prediction of the end-point location that is generated from an efferent copy of the initiated motor command [27, 28]. That is, the difference between the end-point predicted by a first motor plan is based on initial movement conditions and the required end-point after a change in target location generates an error signal, which in turn triggers modulation of the motor command. This process is called an internal feedback loop and it enables the rapid correction of an ongoing movement [27, 28]. Ikudome et al. [29] found that this internal feedback loop is used for movement correction associated with the variable velocity of an oncoming target for coincident timing. The situation was similar in the present study. Therefore, batters might rapidly correct a muscle activation strategy in response to an error signal generated by a difference between the predicted impact time in response to constant target velocity and delayed impact time caused by a decrease in target velocity. Baseball experts can correct movement timing more efficiently than novices when target velocity suddenly decreases [30]. Thus, such swing timing correction might be achieved through repeated batting practice in which batters learn to understand the relationship between impact time and the muscle activation characteristics of the front leg such as the activation pattern, amplitude and/or timing.

Biphasic EMG activation in the VL muscle during DV was visually defined in the present study because the definition of biphasic EMG activation in the front leg during baseball swings is unclear. We showed the characteristics of biphasic activation as: mean latency time from the onset of EMG activation to the first peak (138.7 ± 49.7 ms), mean latency time from the first to the second peak (153.7 ± 71.7 ms) and the mean difference between EMGmax of the first and second peaks (28.9% ± 35.0% MVC). These results provided useful information about how to assess biphasic EMG activation in the front leg during baseball swings.

### Muscle activation characteristics in batters with small timing errors

We also aimed to determine the characteristics VL muscle activation in the front leg of batters with small timing errors. The inter-subject relationships indicated that batters with an early EMG onset and early TP have smaller timing errors than those with later EMG onset and TP in response to unchanged condition. Because the temporal responses were late in response to the unchanged condition (590.1 ± 34.6 ms; that is, impact time was later than target arrival to the impact point), the timing error decreased when the impact time was early. Thus, batters with faster muscle activation onset and peak have faster impact times. As a result, timing errors were small in response to unchanged condition. To accelerate the TP, batters could speed up EMG onset and/or shorten the latency time from EMG onset to TP. Because TP was significantly related to EMG onset but not to latency in response to unchanged condition (Table 3 and Fig 4), TP was dependent on EMG onset in response to this condition. Therefore, batters with small timing errors in response to unchanged condition appeared to use a strategy that accelerated muscle activation onset to reach peak muscle activation faster and achieve an early impact.

Batters with small timing errors in response to DV had a high probability of biphasic EMG activation appearing in response to this condition (Figs 3 and 4, and Table 3). In addition, the timing error was as small as that of batters with a later TP (Table 3 and Fig 4). To delay the TP, these batters could delay EMG onset and/or increase the latency time from EMG onset to TP. Because TP was significantly related to latency time but not to EMG onset (Table 3 and Fig 4), TP was dependent on prolonging muscle activation latency after EMG onset in response to DV. Because the responses were rapid in response to DV (656.6 ± 61.1 ms; that is, impact time was faster than target arrival to the impact point), the timing error became smaller when the impact was later. Therefore, batters with small timing errors in response to DV used a biphasic muscle activation strategy to prolong muscle activation latency after onset to delay reaching the muscle activation peak and achieve a later impact time.

Batters with small timing errors in response to DV had small timing errors in response to all conditions (Table 3). This finding suggested that in coincident timing tasks comprising unchanged and decreased in target velocity, the ability to correct timing in response to a decrease in target velocity is a factor in deciding coincident timing performance in response to all conditions. Enhanced ability to correct for a change in ball velocity might increase general batting performance for pitches including both straight and breaking balls while batting during actual baseball games.

## Conclusion

Swing timing is corrected when the velocity of an oncoming target suddenly decreases by changing the muscle activation strategy of the VL of the front leg from monophasic to biphasic, which delays reaching peak muscle activation time and prolongs impact time.
